# Rac1 selectively binds a specific lamellipodin isoform *via* a noncanonical helical interface

**DOI:** 10.1016/j.jbc.2025.111023

**Published:** 2025-12-06

**Authors:** Tong Gao, Pingfeng Zhang, Alison M. Kurimchak, James S. Duncan, Jinhua Wu

**Affiliations:** Cancer Signaling and Microenvironment Program, Fox Chase Cancer Center, Philadelphia, Pennsylvania, USA

**Keywords:** cell migration, GTPase effector, isoform, lamellipodin, Rac1, RAPH, splicing variant

## Abstract

Lamellipodin (Lpd) is a multifunctional adapter protein that regulates cell migration and adhesion by recruiting Ena/VASP proteins to the leading edge and modulating actin polymerization. The interaction of Lpd and Rho family or Ras family GTPases is crucial for regulating actin dynamics. Contrary to previous assumptions that the main Lpd isoform interacts with Rac1, here we show that strong and specific binding to Rac1 is instead mediated by the short isoform Lpds. This interaction is dependent on Rac1’s GTPase activity and a short insertion (cs2) within the coiled-coil region unique to the Lpds isoform. Structural modeling and mutagenesis analyses further reveal that Lpds engages Rac1 through a noncanonical, single-helix binding mode distinct from the canonical helical-pair configuration. Our results uncover a novel isoform-dependent GTPase:effector binding mode for Rac1-driven actin dynamics, with implications for therapeutic targeting in Rac1-associated cancer progression.

Cell migration and adhesion are mainly controlled by actin polymerization and cytoskeleton remodeling at the leading edge of migrating cells, where lamellipodia and filopodia are formed. Misregulation of these processes leads to many pathological conditions including tumor metastasis and invasion. Among key actin-binding proteins, Ena/VASP (enabled/vasodilator-stimulated phosphoprotein) proteins play central roles in regulating actin dynamics. This activity depends on lamellipodin (Lpd), an MRL (Mig10/RIAM/Lpd) family adaptor protein that recruits Ena/VASP to the leading edge (1, 2).

The MRL proteins are multi-adaptor proteins that share similar domain architectures. In mammals, the two MRL ortholog proteins, Lpd and Rap1-GTP–interacting adaptor molecule (RIAM), both contain a conserved center domain composed of a Ras-associating (RA) and a pleckstrin homology (PH) structural modules, known as the RAPH domain. They also possess an N-terminal talin-binding site (TBS), a coiled-coil (CC) region preceding the RAPH domain, which is followed by several polyproline motifs responsible for interacting with profilin and Ena/VASP (3). The RAPH domain, found in MRL and Grb family proteins, interacts with small GTPase and phosphatidylinositol 4,5-bisphosphate to regulate cell migration and protrusion formation (3, 4, 5, 6). Lpd and RIAM also mediate integrin activation by recruiting talin to the plasma membrane, promoting integrin-mediated cell adhesion (5, 7).

Small GTPases are a family of guanine nucleotide-binding proteins that function as molecular switches to regulate diverse cellular processes in eukaryotes, including cell proliferation, differentiation, and cytoskeletal dynamics. Among all small GTPases, Rac1, a prototype member of the Rho family, regulates cytoskeleton reorganization and lamellipodia formation (8, 9, 10, 11). Recent studies have also indicated Rac1 as a potential therapeutic target due to the important role of Rac1 in driving metastasis and resistance to therapies (12, 13, 14). As a GTPase effector, Lpd has been shown to interact with two small GTPases, M-Ras in the Ras family and Rac1 in the Rho family (15, 16). In cortical neurons, Lpd directly binds to M-Ras and mediates dendritic growth and branching of neuronal dendrite. This process is regulated by plexin through its GTPase-activating protein activity (16). In migrating cells, Lpd localizes to the tips of lamellipodia and filopodia upon Rac1 activation and recruits Ena/VASP protein to the growing protrusions, thus enhancing cell migration by accelerating actin polymerization (1, 17, 18). Lpd also interacts with Scar/WAVE (Suppressor of cAMP Receptor/WASP-family Verprolin-homologous) complex to promote Arp2/3-mediated actin branching and lamellipodial extension (15).

Consistent with its role in cell motility, Lpd is broadly implicated in cancer progression in multiple tumor types. It has been shown to promote glioblastoma invasion, proliferation, and radiosensitivity through epidermal growth factor receptor signaling axis (19). In breast cancer, Lpd promotes metastasis by regulating Ena/VASP proteins and the Scar/WAVE complex (2). In oral squamous cell carcinoma cell lines, upregulated Lpd induced by hepatocyte growth factor/c-Met signaling also promotes oral squamous cell carcinoma cell migration (20). These findings indicate the clinical importance of Rac1-Lpd signaling in cancer.

Although previous studies have shown that CED-10, the *Caenorhabditis elegans* ortholog of Rac1, interacts strongly with MIG-10, the ortholog of Lpd (21), direct interaction of mammalian Lpd with Rac1 has not been explicitly demonstrated. In this study, we identified a specific Lpd isoform, Lpds, that interacts with Rac1 with high affinity. Domain-mapping and mutagenesis reveal a short splicing insertion in Lpds is required for Rac1 binding. Biochemical data and structural analyses further demonstrate that Lpds interacts with Rac1 through a previously uncharacterized interaction, noncanonical helical interface, distinct from the classical β-sheet or helical-pair interactions for typical RA–GTPase interactions (22). Our results reveal an isoform-specific GTPase effector signaling mechanism and a novel GTPase-effector interacting architecture, suggesting a possible Rac1-mediated, isoform-driven metastasis mechanism.

## Results

### Activated Rac1 specifically interacts with one of Lpd isoforms

**Figure 1:**
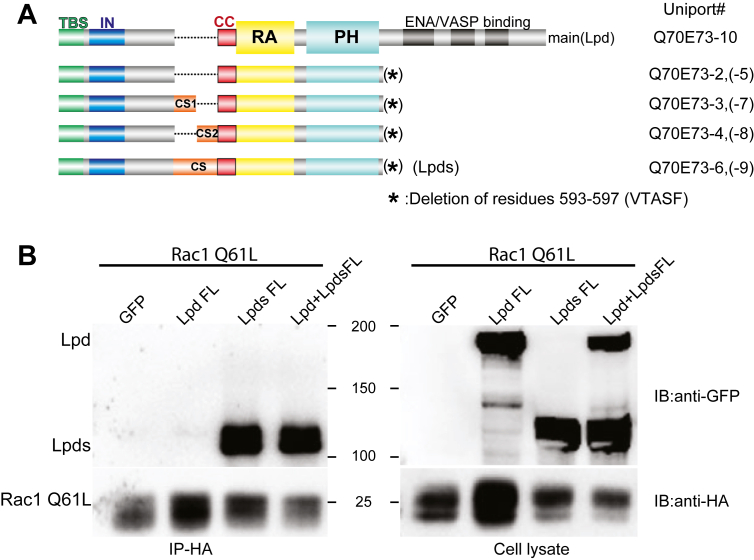
**The Lpds isoform of Lpd has strong binding affinity with activated Rac1 specifically.***A*, schematic representation of Lpd isoforms. A talin-binding site (*green*) is located at the N-terminal end of Lpd and is conserved across all isoforms. The CS is depicted in *orange*, the CC is in *red*, the RA (Ras-associating) domain is in *yellow*, and the PH (pleckstrin homology) domain is in *cyan*. *B*, the co-IP assay of GFP-tagged full-length Lpd and Lpds and HA-tagged constitutively active Rac1 mutant (Rac1 Q61L). Cell lysate and IP samples are detected by Western blot using anti-HA antibody and anti-GFP antibody. CC, coiled-coil region; CS, insertion of cc; IN, autoinhibitory segment; Lpd, lamellipodin; PH, pleckstrin homology domain; RA, Ras-association domain; TBS, talin-binding site.

Although the interaction of *C. elegans* orthologs of Lpd and Rac1 has been demonstrated (21), no significant interaction of mammalian Lpd and Rac1 was detected in our preliminary data. Lpd possesses several isoforms due to alternative splicing (17). The splicing variants often share many conserved functional regions, with some containing additional segments (23). To date, at least nine Lpd isoforms have been identified in human. The main Lpd isoform consists of 1250 amino acids, including an N-terminal TBS, an autoinhibitory segment, a CC region, the RAPH domain, and an unstructured C-terminal Ena/VASP-binding tail (Fig. 1*A*) (5, 6, 17). The other eight isoforms lack the C-terminal Ena/VASP-binding tail. In isoforms Q70E73-3, Q70E73-4, Q70E73-6, Q70E73-7, Q70E73-8, and Q70E73-9, alternative RNA splicing results in two additional helical segments upstream of the CC region. We therefore refer to these insertions as coiled segments (CS), comprising cs1 and cs2.

To examine the impact of the additional CS on Rac1 interaction, we performed co-immunoprecipitation (co-IP) assays to compare the binding affinities of Rac1 with the main isoform of Lpd and a short isoform, Lpds, which contains the CS insertion. Both GFP-tagged full-length (FL) Lpd and Lpds were expressed at similar levels in HEK293 cells and subjected to co-IP with activated Rac1. While no significant interaction was detected with the main isoform, the Lpds isoform co-immunoprecipitated robustly with activated Rac1 (Fig. 1*B*). These findings indicate that Rac1 preferentially binds the Lpds isoform, revealing an isoform-specific interaction.

Previous studies also demonstrate that Lpd can dimerize through an anti-parallel CC interaction involving the CC region (4). This dimerization interaction is crucial for Ena/VASP clustering at the leading edge of protrusions, thus playing an important role in mediating cell migration (1, 24). To investigate if the dimerization through the CC region contributes to Rac1 binding, we co-expressed Lpds, Lpd, and Rac1 in HEK293 cells. Because the Lpds isoform contains an intact CC region, it is capable of forming a heterodimer with the main isoform Lpd. Therefore, if Rac1 binding required a dimeric form of Lpds, both Lpds and Lpd isoforms would be expected to co-elute with Rac1 through Lpd–Lpds heterodimerization. However, only Lpds, but not the main Lpd isoform, was co-immunoprecipitated with Rac1 (Fig. 1*B*). These results indicate that Lpds interacts with Rac1 primarily as a monomer, and the dimerization through the CC region does not contribute significantly to the interaction of Lpds and Rac1.

### Activated Rac1 binding to Lpds requires the CS insertion

**Figure 2:**
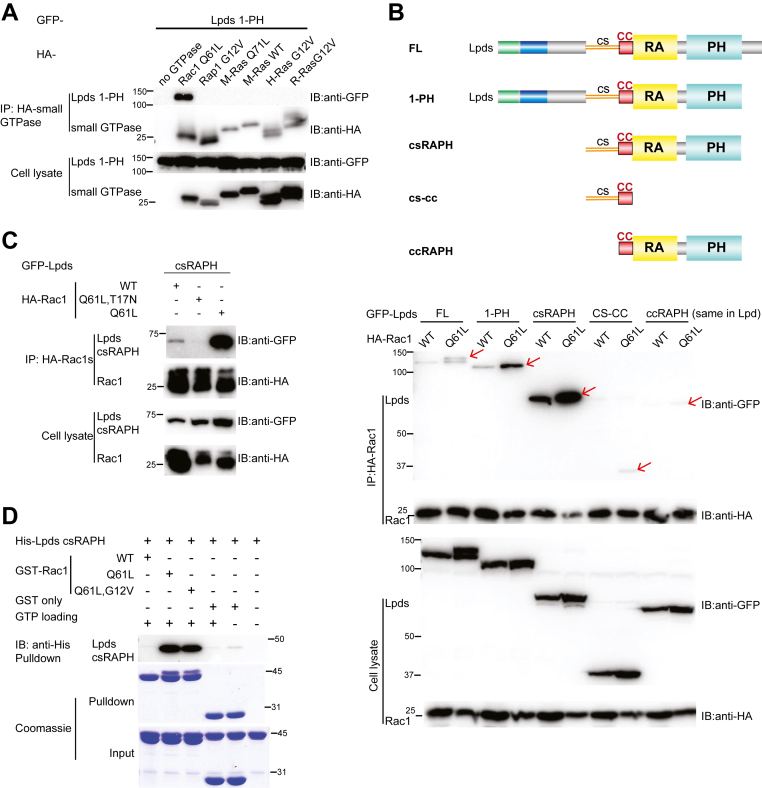
**Rac1:Lpds interaction requires the GTPase activity of Rac1 and the CS insertion of Lpds.***A*, co-IP assay of GFP-tagged Lpds1-PH with HA-tagged Rac1 Q61L, Rap1 G12V, M-Ras Q71L, M-Ras WT, H-Ras G12V, and R-Ras G12V. *B*, co-IP assay of HA-tagged WT Rac1 and Rac1 Q61L with GFP-tagged Lpds FL, 1-PH, csRAPH, cs-cc, and Lpd ccRAPH. The schematic diagrams of the constructs are shown above, with the same domain color codes as in Figure 1*A*. *C*, co-IP assay of HA-tagged WT Rac1, constitutively active Rac1 mutant (Rac1 Q61L), and inactivated Rac1 mutant (Rac1 Q61L/T17N) with GFP-tagged Lpds csRAPH. *D*, pulldown assay of GST-tagged WT Rac1, Rac1 Q61L, and Rac1 Q61L/G12V with His-tagged Lpds csRAPH. CS, coiled segment; Lpd, lamellipodin; PH, pleckstrin homology.

The RAPH module is seen in Grb family and MRL family protein (4, 25, 26, 27). The RA domain typically mediates interactions with small GTPases such as the Ras or Rap GTPases. Previous studies show that both M-Ras and Rac1 can interact with Lpd (15, 16). To investigate whether Lpds can interact with other small GTPases, we examined the binding of Lpds with small GTPases from various Ras families. Among which, M-Ras, H-Ras, and R-Ras are classical members of Ras family cancer therapy (28, 29, 30); Rap1 belongs to the Rap subfamily, which is involved in integrin activation and plays key roles in cell adhesion (4); and Rac1 is an important member of the Rho family that regulates cytoskeleton dynamics (8). A Lpds construct (1-PH) containing the intact RAPH module and the entire sequence upstream of the module was co-expressed with each of the small GTPases in HEK293 cells for co-immunoprecipitation. While Lpds and Rac1 exhibited robust interaction, the co-IP results showed no significant interaction between Lpds and the constitutively active Rap1, M-Ras, H-Ras, or R-Ras (Fig. 2*A*). Our results suggest that Lpds preferentially associates with Rac1, rather than Rap or Ras family members, in modulating specific cellular processes.

We further mapped the Rac1-interaction sites in Lpds by generating a series of truncation constructs. Lpds shares the same sequence with the main Lpd isoform except for the additional CS insertion and the absence of the C-terminal Ena/VASP-binding region. In addition to the FL and 1-PH constructs of Lpds, we generated csRAPH, CS-CC, and ccRAPH constructs. The csRAPH construct contains only the CS, CC, and RAPH segments; the CS-CC construct includes the CS and CC segments; and the ccRAPH construct, which is also present in the main Lpd isoform, contains the CC and RAPH segments (Fig. 2*B*). Co-immunoprecipitation of these constructs with WT or constitutively active Rac1 (Q61L) showed that both the Lpds FL and Lpds 1-PH constructs interacted with Rac1. In contrast, the CS-CC or ccRAPH constructs exhibited minimal interaction. Notably, the csRAPH construct, which contains both the CS insertion and the RAPH domain, displayed the strongest binding to Rac1 among all the Lpds constructs (Fig. 2*B*). These results suggest that the CS insertion is essential for promoting Rac1 binding to Lpds and strengthening their interaction.

We next examined whether the GTPase activity of Rac1 is essential for its interaction with Lpds. We used the csRAPH construct of Lpds to test its interaction with WT, constitutively active (Q61L), and inactive (Q61/T17N) Rac1. Co-immunoprecipitation assays demonstrated that WT Rac1 binds to Lpds csRAPH, while the constitutively active Rac1 (Q61L) exhibits much enhanced binding with Lpds csRAPH. In contrast, the interaction with Lpds is abolished with the inactive Rac1 mutant (Q61L/T17N) (Fig. 2*C*). To rule out the possibility of indirect or mediator-assisted interactions through other cellular components, we purified His-tagged csRAPH and GST-tagged Rac1 proteins and performed *in vitro* pulldown assays. GTP-loaded, constitutively active Rac1 constructs, Q61L and Q61L/G12V, exhibit markedly stronger interaction with csRAPH of Lpds than WT Rac1(Fig. 2*D*). These results confirm that Rac1 directly interacts with the csRAPH region of Lpds in a GTPase activity–dependent manner.

### The Lpds:Rac1 complex adopts a non–β-sheet binding mode distinct from the Rap1:RIAM interaction

**Figure 3:**
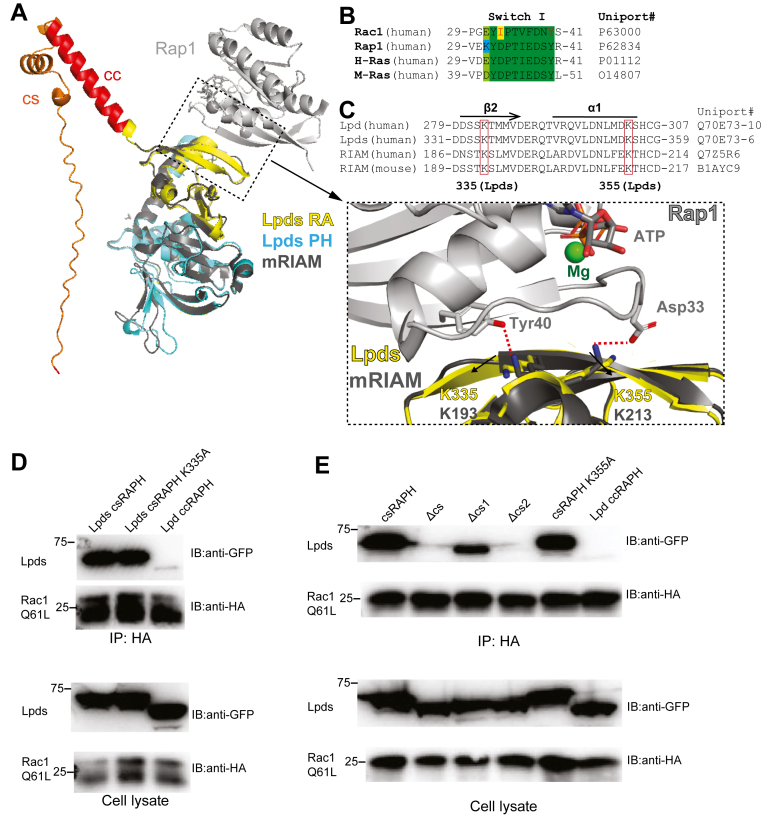
**Lpds binds Rac1 through a non–β-sheet interface distinct from the RIAM:Rap1 interaction.***A*, structural alignment of the AlphaFold-predicted, Lpds alone model with the crystal structure of the RIAM:Rap1 complex. Lpds domains are colored as follows: CS insertion (*orange*), CC (*red*), RA domain (*yellow*), and PH domain (*cyan*). The RIAM:Rap1 complex is shown in *gray*. The zoomed-in panel (*bottom right*) highlights key salt bridges in the RIAM:Rap1 interface (Asp33:Lys213 and Tyr40:Lys193). The corresponding residues in Lpds (Lys335 and Lys355), selected for mutagenesis, are labeled. *B*, sequence alignment of the switch I region from Rac1, Rap1, H-Ras, and M-Ras. Conserved residues are highlighted in *green*. Rac1 residues Ile33 and Tyr40, corresponding to salt bridge–forming residues in Rap1, are marked in *red*. *C*, sequence alignment of Lpd, Lpds, and RIAM, with lysine residues involved in Rap1:RIAM interaction highlighted in the boxes. Lpds numbering is based on the sequence including the CS insertion. *D*, co-IP assay of HA-tagged Rac1 Q61L with GFP-tagged Lpds csRAPH, csRAPH K335A, and Lpd ccRAPH. *E*, co-IP assay of HA-tagged Rac1 Q61L with various truncations of GFP-tagged Lpds csRAPH and csRAPH K355A. CC, coiled coil; CS, coiled segment; Lpd, lamellipodin; PH, pleckstrin homology; RA, Ras-associating; RIAM, Rap1-GTP–interacting adaptor molecule.

We then probed the binding mode of Rac1 with the csRAPH region of Lpds. Since crystallization attempts of the Rac1:csRAPH complex or their fusion protein were unsuccessful, we performed structure prediction of csRAPH using Alpha-Fold (31). The predicted structure was then aligned with the RAPH structure of RIAM from the RIAM:RAP1 complex crystal structure (PDB ID: 4KVG) (25). In the predicted model, the CC region and the RAPH module are consistent with the previously reported crystal structure of the CC-RAPH fragment of the main Lpd isoform (4). The N-terminal half of the CS insertion is largely unstructured, while the C-terminal half of the CS forms two short α-helices that contact with CC region, likely stabilizing its helical configuration (Fig. 3*A*). The RAPH module of Lpds closely resembles that of RIAM in the RIAM:Rap1 complex, with an RMSD of 0.7-Å over all atoms.

Interestingly, structural modeling suggests that if the Rac1:Lpds interaction resembles a canonical antiparallel β-sheet interaction of typical RA:GTPase interactions as observed in the Rap1:RIAM complex, no direct contact involving the CS region would be expected (Fig. 3*A*). In the Rap1:RIAM complex structure, two hydrogen-bond interactions are observed between RIAM-Lys193 and Rap1-Tyr40, and between RIAM-Lys213:Rap1-Asp33. While both Asp33 and Tyr40 are highly conserved across Ras family GTPases, only Tyr40 remains conserved in the Rho family (Fig. 3*B*). Although the corresponding lysine residues are conserved in Lpds as Lys335 and Lys355 (residue numbering including the cs insertion) (Fig. 3*C*), substitution of Asp33 by isoleucine in Rac1 prevents the formation of the signature Asp-Lys hydrogen bond (25), suggesting that the Rac1:Lpds interaction adopts a noncanonical RA:Ras binding mode. To test this, we mutated Lys355 in Lpds, which corresponds to RIAM-Lys213 in RIAM that forms the hydrogen bond with Rap1-Asp33 in the Rap1:RIAM complex. Lpds K355A exhibits similar binding affinity to Rac1 as WT-Lpds, supporting the absence of the signature Asp-Lys or any compensatory hydrogen bond in the Rac1:Lpds interaction (Fig. 3*D*). Moreover, we generated a K335A mutation, which also had minimal effect on Rac1:Lpds interaction, further supporting that the Rac1:Lpds interaction adopts a noncanonical RA:GTPase binding mode distinct from the RIAM:Rap1 interaction (Fig. 3*E*).

### Leucine clusters in the CS region are critical for the noncanonical Rac1:Lpds interactions

**Figure 4:**
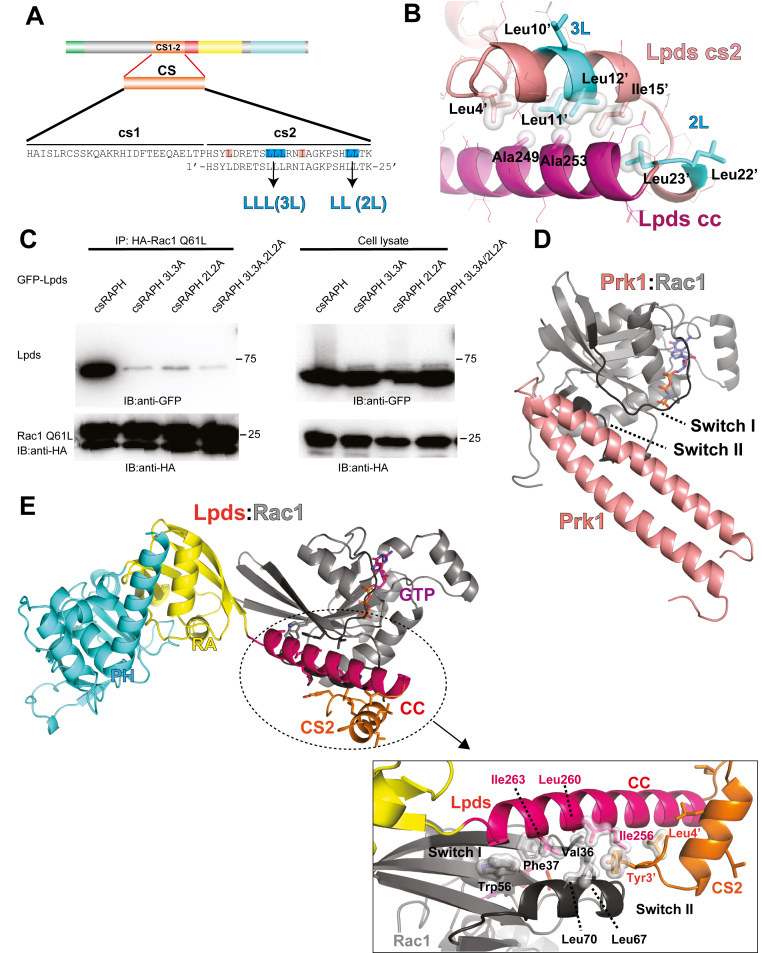
**Structure prediction and co-IP assay suggest that the cs2 insertion stabilize the CC region *via* hydrophobic interaction.***A*, amino acid sequence of the two Lpd splicing insertions, cs1 and cs2, are shown. The cs2 insertion residues are numbered 1′ to 25’. Two hydrophobic leucine clusters, LLL (3L) and LL (2L), in the cs2 insertion are highlighted in *cyan*. Leu4′ and Ile15′ of cs2 are colored in *pink*. *B*, predicted interface between the cs2 insertion (in *pink*) and CC region (in *red*). Hydrophobic residues contributing to the cs2:CC interaction are shown in surface representation. Residue numbers of CC are based on the Lpd main isoform to avoid confusion. *C*, co-IP assay of HA-tagged Rac1 Q61L and GFP-tagged Lpds csRAPH, Lpds csRAPH 3L3A, Lpds csRAPH 2L2A, and Lpds csRAPH 3L3A/2L2A. *D*, structure of Rac1 (in *gray*) in complex with the helical-pair for Prk1 (in *pink*). *E*, structure Lpds:Rac1 complex predicted by AlphaFold3. *Upper*: Lpds is colored as indicated in Figure 1*A*. Rac1 is colored in *gray*. *Lower*: Interface between Lpds-cs2-CC and Rac1. Switch I and switch II regions of Rac1 are colored in *dark gray*. Residues forming the hydrophobic interaction interface are shown in surface representation. CC, coiled coil; CS, coiled segment; Lpd, lamellipodin.

The CS insertion in Lpds includes two alternative splicing segments, cs1 and cs2. Depending on the splicing product, each isoform may contain cs1 alone (isoforms #3, #7), cs2 alone (isoforms #4, #8), both cs1 and cs2 (CS, isoforms #6, #9), or neither (isoforms #2, #5, #10) (Fig. 1*A*). The highly flexible N-terminal extension prevents the crystallization of the Lpds. To explore the structural features of the cs1 and cs2, we first performed Alphafold prediction for the FL Lpds alone. Although secondary structure prediction suggests a weak helical tendency in cs1, structure prediction indicates a largely unstructured cs1 region (Fig. 3*A*). In contrast, cs2 is predicted to form one -helix and a short helical loop that contain two leucine clusters, Leu10′-Leu11′-Leu12′ (3L) and Leu22′-Leu23′ (2L) (residue numbering within the cs2 insertion only) (Fig. 4*A*). The cs2 region had an average pLDDT of 83.6, indicating high confidence in backbone prediction. To test the impact of cs1 and cs2 on Rac1 binding, we generated a series of deletion constructs derived from the csRAPH segment by removing either cs1 (Δcs1) or cs2 (Δcs2), or both (ΔCS), and performed co-IP assays with constitutively active Rac1 (Fig. 3*E*). Removal of cs1 had minimal impact on Rac1 binding, whereas removal or cs2 or removal of both cs1 and cs2 completely abolished the interaction between Lpds and Rac1, indicating that cs2 plays an essential role in mediating Rac1 binding.

Leucine clusters are often involved in hydrophobic interaction and helix stabilization. We then investigated the role of these leucine clusters in Rac1 binding. In the predicted model, the 3L and 2L are positioned near Leu4′ and Ile15′ in the cs2 and Ala 249 and Ala253 in the CC region (Fig. 4*B*), suggesting that they help stabilize the CC helix *via* intramolecular hydrophobic packing. To test the impact of the Leu clusters, we generated 3L3A (LLL to AAA) and 2L2A (LL to AA) mutations. Co-IP assays using the constitutively active Rac1-Q61L demonstrate that the disruption of either clusters or both significantly diminishes Rac1 binding (Fig. 4*C*). These results indicate that both leucine clusters contribute to either direct interaction with Rac1 or stabilizing the structural configuration required for the interaction.

### Structure prediction reveals a noncanonical Rac1:Lpds interaction mediated by a cs2-stabilized helical surface

Extensive crystallization trials of the Lpds:Rac1 complex did not yield diffracting crystals, likely due to the structural plasticity of the cs2 insertion. We then performed Alphafold3 structure predictions for GTP-bound Rac1 in complex with various Lpds constructs. All predicted models consistently reveal a conserved interface between the cs2-CC region of Lpds and the GTP-bound Rac1, in strong agreement with our biochemical data. The representative complex model achieved an inter-protein predicted TM-score score of 0.83 and minimal interchain predicted aligned error values ranging from 1 to 4 Å, suggesting high confidence in the predicted chain–chain interfaces. These findings suggest that the predicted interaction of the Lpds:Rac1 complex is structurally robust and biologically reliable.

The predicted Lpds:Rac1 complex structure reveals a previously unrecognized binding mode of GTPase:effector. Most of the small GTPases in the Rho and Ras family engage their effector proteins *via* a canonical β-sheet interaction with the RA of Ras-binding domain or a helical-pair interaction. In the classic helical-pair interaction such as the Rac1:Prk1 complex (PDB ID: 2RMK, Fig. 4*D*), a pair of Α-helices from the effector contact the switch I and switch II regions of the GTPase. The helical pairs are stabilized through CC interaction or other interhelical packing, forming an extensive interface with the target GTPase. In contrast, although all Lpd isoforms contain a CC region that mediates an intermolecular CC in a homodimer formation (4), no significant interaction is observed between Lpd main isoform and Rac1, indicating that the Lpds:Rac1 interaction is not mediated by the canonical helical-pair binding mode. Instead, structure prediction suggests that the CC region of Lpds is stabilized by the cs2 insertion, forming a hydrophobic surface that directly interacts with the corresponding hydrophobic surface between switch I and switch II regions in the active Rac1(Fig. 4*E*). The interaction is mediated by residues Val36, Phe37, Leu67, and Leu70 in the switch regions and residue Trp56 from Rac1 and residues Leu256, Leu260, Ile263 in CC (Fig. 4*E*). This interface is further reinforced by Tyr3′ and Leu4′ residues from the cs2 insertion. These structural features also differ from the canonical helical-pair interacting modes observed between Rho family GTPases and their effectors. Thus, in contrast to the classical β-sheet and helical-pair binding modes, the Lpds:Rac1 interaction reveals a previously unrecognized, splicing insertion-dependent, single-helix binding mode.

## Discussion

Small GTPases are ubiquitously expressed across tissues and play central roles in regulating cell growth, differentiation, and migration. Rho family GTPases, including Rac1, are primarily associated with cancer metastasis due to their regulation of cytoskeletal dynamics. Multiple cancer-associated mutations in Rac1 have been identified, including the melanoma hotspot mutation P29S (32). Rac1-P29S increases the flexibility of switch I (33), enhances nucleotide exchange (34), and promoted melanocyte proliferation and migration (35). Moreover, the P29S mutation also contributes melanoma resistance to RAF inhibitors (36) and may alter PD-L1 expression (32). Another mutation, A159V, has been shown to be associated with head and neck squamous cell carcinoma (37, 38). Overall, Rac1 is emerging as a promising therapeutic biomarker in selected cancers.

Our data suggest that the cs2 insertion in the Lpds isoform is required for Rac1 binding by stabilizing a hydrophobic interface between the CC region and Rac1. However, the CS–CC region alone is insufficient to mediate strong interaction, suggesting that the RA–PH tandem domain also contributes to Rac1 association. Mechanistically, the RA–PH module may facilitate this interaction by stabilizing the helical conformation of the upstream CC region, as previously reported for other MRL family proteins (4, 6), and by interacting with phosphoinositides to anchor Lpd at the plasma membrane. This membrane localization promotes association with membrane-anchored Rac1 and supports lamellipodia formation and actin remodeling. Notably, this cs2 insertion is absent in the Lpd main isoform. Conversely, the Ena/VASP-binding region in the Lpd main isoform is also absent in the Lpds isoform. Our data suggest that Lpd contributes to Rac1-mediated actin regulation through two complementary mechanisms: Lpds engages Rac1 signaling *via* the cs2-enhanced single-helix binding mode, while the Lpd main isoform regulates actin polymerization machinery *via* Ena/VASP recruitment. These distinct functions may be coordinated by Lpd dimerization or by interactions with other actin-regulatory complexes.

Previous studies have revealed the expression of Lpds and Lpd in fibroblasts (17). To investigate the role of Lpd in cancer, we analyzed the expression levels of Lpd isoforms across a panel of cancer and normal cell lines (Fig. S1). We found that both Lpd and Lpds expression levels are markedly elevated in multiple cancer cell lines, whereas their expression is very low or undetectable in normal tissues, such as adult brain, and in normal fibroblast or epithelial cells such as IMR90 and RPE1. Notably, the ratio of Lpds to total Lpd is also increased in most cancer lines (*e.g.*, HeLa, H446, and PNX001), suggesting that cancer cells not only upregulate total Lpd expression but may also favor production of the Lpds isoform. This observation supports a potential isoform-specific regulation mechanism in malignancy. The discovery of a single-helix binding motif in the Lpds–Rac1 complex presents a previously unrecognized structural principle for GTPase-effector recognition. Such single-helix interfaces have recently been recognized as attractive templates for developing stapled-peptide inhibitors that modulate intracellular signaling (39, 40). This isoform-specific expression pattern suggests that Lpds may serve as a biomarker of metastatic activity and a candidate for therapeutic targeting. Further work is needed to examine whether Lpds supports tumor-specific Rac1 signaling and whether its inhibition can reduce cancer cell migration without affecting normal cells.

## Experimental procedures

### Plasmid construction

Lpd constructs were subcloned into a modified pET28a vector with a hexahistidine-tag (His6-tag). The Rac1s were subcloned into pGEX-5X-1 vector with a GST-tag. Lpd was subcloned into an EFEP-C3 vector, and Rac1 was subcloned into pCGN vector for expression in HEK293 cells. Point mutations were generated with a site-directed mutagenesis according to the QuikChange site-directed mutagenesis manual.

### Protein purification

The His6-tagged proteins and GST-tagged proteins were expressed and purified according to the procedures described previously (25, 41). Briefly, *Escherichia coli* BL21(DE3) was transformed with pET28a-Lpd constructs or pGEX-Rac1 constructs. The positive colonies were cultured in LB medium with 50 μg/ml kanamycin or 80 μg/ml ampicillin in shaking flasks (200 rpm) at 37 °C. To induce protein expression, 0.2 mM IPTG was added to the flasks when the absorbance at 600 nm (A600) reached 0.7. For His6-tagged protein production, the flasks kept shaking overnight at 16 °C and for GST-tagged protein production, the flasks kept shaking at 37 °C for 3H. The bacteria were collected by spinning down at 2200 rpm for 30 min and were resuspended with 20 mM Tris pH 7.5, 500 mM NaCl for His6-tagged proteins and 20 mM Tris pH 7.5, 100 mM NaCl for GST-tagged proteins. EmulsiFlex-C3 was used to homogenize the resuspension, and the supernatant was applied to HisTrap FF columns (Cytiva) or GSTrap HP columns (Cytiva) for purification using ÄKTA Purifier (Cytiva).

### Co-immunoprecipitation

GFP-tagged Lpd proteins and HA-tagged Rac1 proteins were co-expressed in HEK293 cells. The cell lysates were rotating with α-HA (sigma, H9658-.2 ML)-conjugated Surebeads magnetic beads (161-4023, Bio-Rad) for 2H at room temperature. The supernatant was removed, and the beads were washed with PBS+0.1% Tween 20 three times. Samples were eluted by incubating the beads with glycine (20 mM, pH 2.0) for 5 min at room temperature. After mixing with Laemmli sample buffer (S3401-10VL) and heated to 95 °C for 10 min, the samples were applied to Western blotting. Anti-GFP (Sigma, G1544, 1:2000) and anti-HA (sigma, H9658-.2 ML, 1:2000) were used for the detection of GFP-tagged Lpd proteins and HA-tagged Rac1 proteins. A FluorChem E System (Proteinsimple) with a charge-coupled device camera was used to expose the Western blotting membrane.

### GST pull-down

Purified GST-Rac1 proteins (100μl, 1 mg/ml) were incubated with 2 mM EDTA on ice for 30 min then incubated with 5 mM GTP and 20 mM MgCl2 on ice for 30 min. His6-Lpd proteins (100μl, 1 mg/ml) were mixed with GTP-loaded GST-Rac1 proteins in binding buffer (50 mM Tris, pH 7.5, 100 mM NaCl, 2 mM DTT). The protein mixture was rotating for 1 h at 4 °C and then incubated with binding buffer–equilibrated glutathione agarose beads (Invitrogen, G2879) on a rotator for 1 h at 4 °C. The beads were washed with binding buffer three times after the supernatant is removed. Bound proteins were eluted with 40μl elution buffer (50 mM Tris, pH 7.5, 100 mM NaCl, 2 mM DTT, 10 mM reduced glutathione). Coomassie staining and Western blotting were used to evaluate the result after samples were applied to SDS/PAGE. Anti-His (Sigma, H1029-100UL, 1:2000) was used for the detection of His6-tagged THD proteins.

### Alphafold3 structure prediction

Structural prediction of Lpds alone and the Lpds:Rac1 complexes were performed using Alphafold3 (AlphafoldServer.com) (42). Human Lpds and Rac1 sequences were used as input protein sequences, for Lpds:Rac1 complexes; GTP is also included as a ligand for the prediction in the multimer mode for modeling heteromeric complexes. Model confidence was evaluated by predicted local distance difference test, the interprotein predicted TM-score, and the predicted aligned error. Structure analyses and figure preparation were carried out by Pymol.

## Data availability

All data supporting the findings of this study are included within the article and supporting information.

## Supporting information

This article contains supporting information.

## Conflict of interests

The authors declare that they have no conflicts of interests with the contents of this article.
