# Supplementary material for: Rac1 selectively binds a specific lamellipodin isoform via a noncanonical helical interface
Source: J Biol Chem. 2025 Dec 6;302(1):111023. doi: 10.1016/j.jbc.2025.111023 (PMC12797038; doi:10.1016/j.jbc.2025.111023)
Supplement: Fig. S1 [file mmc1.pdf]

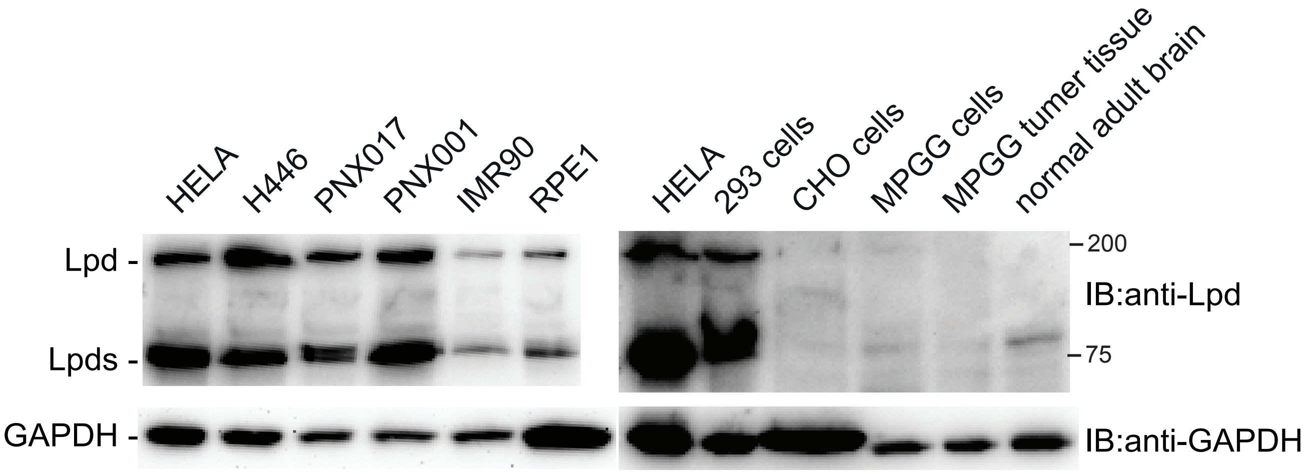

Expression level of isoforms of Lpd in different cells lines.
Lpd isoforms expression in HeLa (cervical cancer), H446(lung cancer), PNX001(pancreatic cancer), PNX017(pancreatic cancer), IMR90 (lung), RPE1(epithelial), 293 (kidney cell),CHO (Chinese hamster ovary cell), normal brain tissue and MPGG tumor cells and tissue.

Supplymental Figure
